# Human-animal entanglements in bushmeat trading in Sierra Leone: An ethnographic assessment of a potential zoonotic interface

**DOI:** 10.1371/journal.pone.0298929

**Published:** 2024-03-28

**Authors:** Jack Jenkins, Wahab Lawundeh, Tommy Hanson, Hannah Brown

**Affiliations:** 1 Department of Anthropology, Durham University, Durham, United Kingdom; 2 Department of Sociology and Social Work, Njala University, Bo, Sierra Leone; University of Ibadan Faculty of Veterinary Medicine, NIGERIA

## Abstract

‘Bushmeat’ markets are often portrayed as chaotic spaces where exotic wild animals are sold. They are hypothesized to be important sites for zoonotic disease transmission, given the prolonged and intense nature of the cross-species encounters that occur within them. Whilst such markets have received some attention from researchers, rich qualitative descriptions of everyday practices in these markets are rare. Depictions of wild animal markets as sites for potential viral amplification often rely on exoticizing assumptions and narratives rather than actual evidence, and in some cases are based more on ideology than on science. We provide an in-depth ethnographic account of two bushmeat markets in Bo, Sierra Leone. Our analysis goes beyond common assumptions that zoonotic risk is located solely in the knowledge and behaviours of traders. Our account sheds light on the modes of touch, closeness and contact that shape this hypothesised zoonotic interface, outlining the possible risks to different people who use and spend time in the market. We found that inadequate infrastructure and sanitation facilities created risks of zoonotic disease transmission for diverse actors including traders, customers, children, and the wider public. Butchering and trading practices frequently resulted in people directly and indirectly encountering animal fluids. We also discuss how public health management of these markets focused on individual behaviours rather than on improving conditions. Urgent sanitary reform and infrastructure upgrades in these sites that support the economic needs of traders could encourage voluntary compliance with biosafety measures amongst traders seeking to balance responsibilities to family and public health. Our study reveals the value of moving beyond exoticized narratives about bushmeat markets to yield situated insights for reducing risk at this interface.

## 1. Introduction

Across parts of Africa, so-called ‘bushmeat’ markets and butcheries are hypothesised to be intensive sites for human-animal encounters and for the possible spread of zoonotic diseases. Markets are a crucial node in the wider trade in bushmeat, a trade that has become an important topic of focus among public health officials and environmentalists both for the risk of the transmission of emergent zoonotic diseases it poses [1, 2], and for the damaging impact that the trade has on conservation efforts [3, 4].

People risk being exposed to zoonotic pathogens at various points in the bushmeat commodity chain, for example while hunting or transporting bushmeat to markets. However, it is during the act of butchering animals that the risk of transmission is highest [1]. LeBreton et al. [5] explain that while butchering wild animals, individuals risk being sprayed with blood during cutting, coming into contact with blood when handling meat, being injured by claws and bones, or being cut when using cutting utensils. When in contact with blood and body fluids during butchering, they face the risk of contracting infections through open wounds or through injuries from knives and bone fragments [5]. Most cuts are minor enough that the butcher does not stop work to tend to the wound [6]. Because self-cutting leaves open wounds where bloodborne pathogens can enter, the act of cutting oneself while butchering creates a vulnerability to disease transmission that lasts for some time. The question of who is responsible for butchering animals varies across contexts. Research conducted in Laos found most live animals purchased at markets were processed (i.e., killed and butchered) by consumers themselves [7]. In Sierra Leone, the focus of this study, activities of bushmeat traders typically include butchering, preparing, and selling bushmeat [8]. Another quantitative study conducted with hunters and bushmeat traders in 37 locations across southern Sierra Leone (the location of our field sites) found that self-cutting was more frequent among individuals who engaged in trading compared to individuals who hunted, with 38% of traders indicating that they cut themselves on a regular basis during butchering [8]. In Central and West Africa, bushmeat traders are usually women [5, 9–12] and women are much more likely than men to be involved in the butchering of wild animals [5]. Because of the butchery tasks associated with trading, women have been found to be especially vulnerable to zoonotic disease transmission [8].

Since 2019, in the context of the COVID-19 pandemic, when so-called ‘wet markets’ selling meat and wild animals were repeatedly implicated in stories about the emergence of the virus, wild animal markets and the related trade in bushmeat have regained attention at the global level [13]. The term ’wet market’ is used to describe a wide diversity of different types of markets selling fresh meat and produce in East Asia and emerged in distinction from markets selling ‘dry’ and packaged goods such as textiles [14]. Anthropologists and other critical commentators discussing the COVID-19 origin stories linked to the Wuhan wet market have pointed to the ‘Orientalist’ nature of many accounts, arguing that during the pandemic the Western media portrayed such markets as “emblems of Chinese otherness: chaotic versions of oriental bazaars, lawless areas were animals that should not be eaten are sold as food, and where what should not be mingled comes together (seafood and poultry, serpents and cattle)” [14, 15]. For these scholars, what is at issue is not simply a case of racist tropes occluding understandings of the diversity and actual workings of these markets (although this is an important issue), but a wider problem of knowledge politics. Christos Lynteris [16] has described the search for an original ‘spillover’ event as entailing an ‘ontological imperative’, where viral emergence ‘must be’ the result of ‘exotic’ forms of human-animal contact. This ontological imperative can be extended to other frequent assumptions about the origin of the COVID-19 virus, that there ‘must be’ a single sudden viral leap from animals to humans rather than a pathogenic interface consisting of multiple forms of multispecies entanglement, viral contact and sharing [17]. In these assumptions, sites of risk ‘must be’ those marked by the exotic consumption of wild animals rather than the human-animal entanglements created through practices of everyday labour that are enmeshed in global capitalist economies [18].

An ontological imperative that marks much of the literature on bushmeat markets in Africa is that zoonotic disease risk ‘must be’ the result of the behaviours and perceptions of market workers and their (inadequate) understandings of risk. Whilst we are critical of the limitations of the narrow focus of some of this literature (see below), taken together it nevertheless allows us to make some important generalisations about the organisation of bushmeat markets in Central and West Africa. In the paragraphs that follow, we summarise this literature before outlining the added benefits of taking an ethnographic approach to studying this risk interface.

Across the region, bushmeat is often valued as a tasty and culturally important delicacy, but also as a healthy, nutritionally beneficial food [5, 8, 11, 12]. In the Democratic Republic of the Congo (DRC), wild animals are commonly viewed as ‘pure’ and ‘natural’, unlike domestic animals which have been ‘tarnished’ by human interference [19]. Given these positive associations, traders and butchers may not believe that live animals or animal meat can transmit diseases to humans, or that they are themselves at elevated risk for illness [6]. Such beliefs may also arise because people have never personally seen or recognized such transmission occurring despite common and frequent human-wildlife interactions across multiple generations [6]. Therefore, despite multiple disease outbreaks globally and well-documented zoonotic diseases associated with bushmeat, perceived health risks are typically low among bushmeat handlers [13]. In Sierra Leone [8], Ghana [11], Nigeria [20, 21], Laos [7, 22], the Democratic Republic of the Congo [23], and Cameroon [6], risk perception related to zoonoses has been found to be low among highly exposed groups like hunters and vendors. Trefon [12] notes that in Central Africa, a prevailing belief that life trajectories are predetermined, rooted in and reinforced by Christianity, can lead individuals to perceive sickness or death as the will of the supernatural and therefore to run the risk of infection. In Nigeria [24] and Laos [7], people with higher levels of education were more likely to be aware of zoonotic disease risk, suggesting that there may be opportunities to increase knowledge of wildlife diseases through awareness and engagement campaigns. However, in Sierra Leone education was not found to have a significant influence on knowledge about zoonotic infection risks amongst bushmeat handlers [8].

In most contexts, bushmeat traders do not generally perceive their activities to carry a particular risk of disease transmission. This insight likely explains why traders rarely deploy strategies to mitigate zoonotic disease risk, for example by using personal protective equipment (PPE) such as gloves, face masks, or aprons. Alhaji et al. [21] find market vendors’ use of preventive health behaviours including washing or sanitising hands after touching wildlife (22.1%) and using PPE (15.8%) were low, but not non-existent. According to Ozioko et al. [20], traders in Southeast Nigeria are for the most part unconcerned about bushmeat-associated zoonoses and are unlikely to take proper precautions. In their study of social and behavioural risks of emerging viral threats in Cameroon, Saylors et al. [23] find that most market workers and hunters do not consider the use of PPE to be important. Several mentioned that gloves are not a feasible protective measure–hospital-style gloves are too thin to protect against anything, and larger gloves used for heavier tasks are too cumbersome for the work they do [23]. In the DRC, many butchers wear clothes for market work that are kept separate from home clothes, but PPE such as gloves, masks, and boots are rarely used by bushmeat vendors or butchers [23]. Mistrust in government messaging may preclude belief in zoonotic risk and the uptake of risk mitigation strategies [19]. Interestingly, those who do perceive contact with wildlife to be risky do not always attempt to mitigate that risk. In a study from Cameroon where most people believed contact with bushmeat fluids to be risky, just 4 percent of hunters and 2 percent of people reporting butchering indicated that they took precautions while carrying out those tasks [5]. However, Alhaji et al. [24] found significant use of mitigation measures against Ebola virus infections by Nigerian bushmeat handlers, including cleaning and disinfection of equipment and surroundings and the use of PPE.

A related set of concerns emerging across the literature relates to overcrowding, inadequate hygiene, and improper storage of animals, faeces, and disposal of carcasses [25]. Bushmeat markets are frequently characterised by a lack of sanitation facilities and infrastructure. In Cameroon, Saylors et al. [6] observed that after butchering, individuals usually just wiped their hands dry with a rag, newspaper, or grass rather than rinsing them. Insufficient washing of hands, tools, and work surfaces is encouraged by inadequate access to water sources, which may require water to be carried from sources outside the market [6]. The risk of human infection with pathogens was found to be higher in markets where butchering occurs due to unsafe disposal of blood and entrails on the ground, and a lack of cleaning of butchery instruments and worktables [26].

Existing literature thereby begins to provide a rich picture of some shared features of bushmeat markets in different African settings. However, beginning from the ontological assumption that risk ‘must be’ due to the knowledge, practice and behaviours of butchers/traders entails a significant risk; local logics become viewed simply as dangerous and in need of correction, rather than the basis on which to build solutions that are inclusive of traders’ needs and locally practical [cf. 27]. Moreover, such a premise tends to locate risk within specific individuals, overemphasising their relative importance and agency within complex interactive landscapes of differentially distributed risk. This individualised lens also often obscures other relevant structures and contexts, in particular hygiene controls and facilities that traders engage with. It similarly under-accounts for the risk faced by the relational dimensions of bushmeat trading, including to people whom traders are in close contact with during their work, and others who use the market who may also come into contact with bodily fluids of wild animals. Two key gaps that are revealed in our review are (1) that studies have rarely explored the risk posed to customers and others present at bushmeat/wet markets and (2) most studies fail to consider how people modify their behaviour in light of public health ordinances and controls. A key contribution was made to the first question by Pruvot et al. [7], who demonstrated high rates of direct contact between bushmeat and people visiting markets (on average 7 contacts per animal per hour), revealing animal-human contact at wet markets as a potential underestimated route of disease transmission in the bushmeat system. Rarer species seem to attract particularly high numbers of contacts, so the disproportionately high number of contacts they receive may have a significant impact on transmission analogous to super-spreading events [7]. The second question was under-researched across the literature. The few studies that spoke about how vendors modify their behaviour in light of enforcement actions in bushmeat markets did not speak about enforcement of public health measures specifically, but enforcement actions more generally, including enforcement of conservation legislation [6, 7, 26].

**Fig 1:**
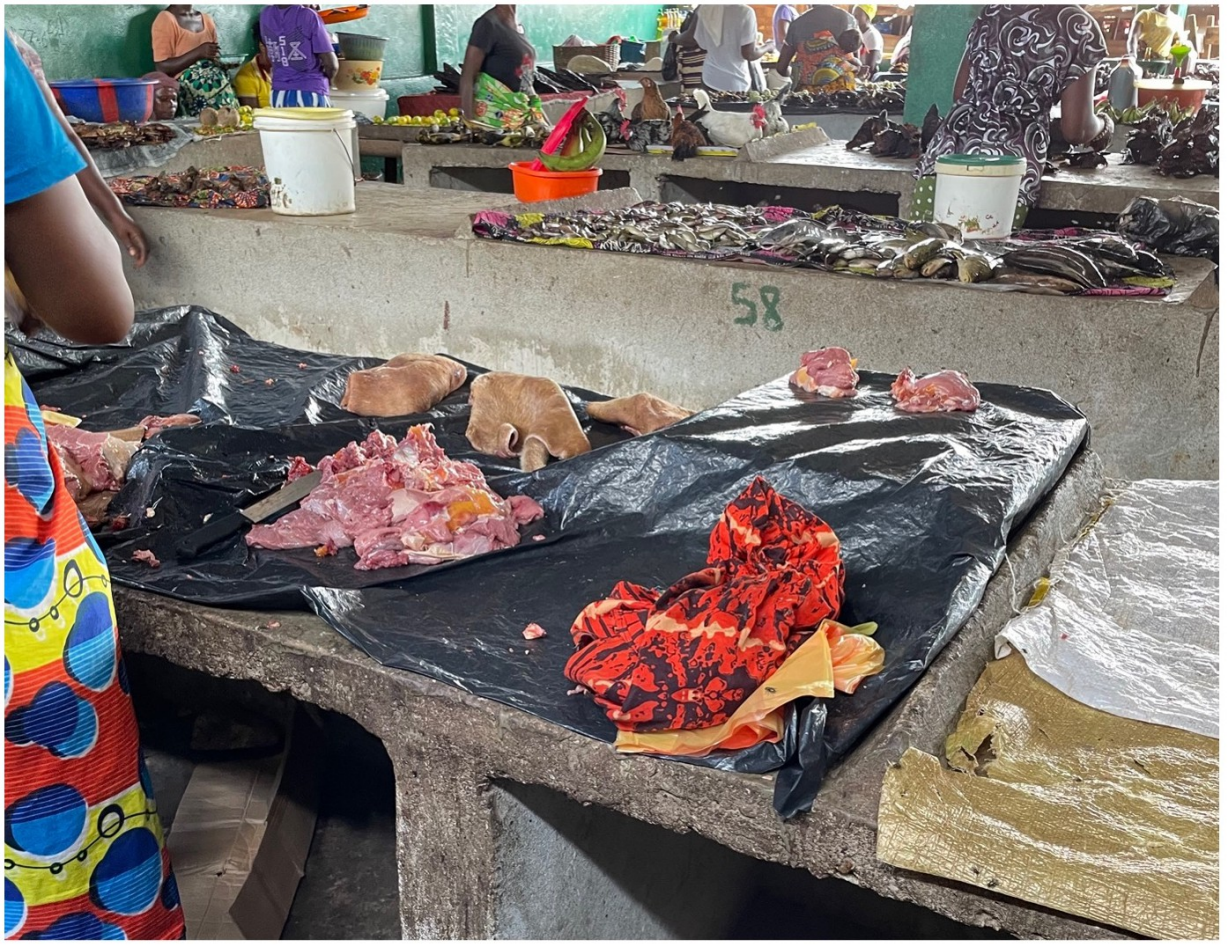
A photo of a market in Southern Sierra Leone where bushmeat is being sold alongside fresh and dried fish, fruits, and live chickens.

In the case of bushmeat markets, what is needed, we argue, is a better understanding of these markets as sites of potential zoonotic amplification that is based on robust empirical analysis about the way markets work that opens new areas of attention rather than being narrowed by forms of received wisdom that precondition our analytic gaze. After all, as Roe et al. [28] point out about ‘wet markets’, it is important to understand that bushmeat markets are often simply food markets which sell a range of fresh produce: fruit and vegetables, fish, livestock, and sometimes wildlife (see Fig 1), and meet a number of important livelihoods and economic needs which shape wild animal encounters in important ways. Ethnographic approaches offer an important way of holding the mundane, everyday aspects of bushmeat trading in view and understanding how risk is distributed across the range of different people implicated in these quotidian practices, whilst opening up attention to a wider set of relevant relations, interactions, and structures that shape the conditions of this potentially pathogenic interface [29]. Our own material is an example of how ethnographic approaches can reveal new dimensions to practices of bushmeat trading that matter for understanding markets as sites of potential zoonotic emergence and mitigating transmission.

Much of the literature we have reviewed is quantitative in design. Our insights extend the few contributions that have taken a more open or ethnographic approach to these issues [6, 12, 19] to explore who is involved in the different stages in the preparation and sale of meat including customers, other market vendors, and passers-by; what kinds of activities within the market might put different people at risk; what risk-mitigation strategies sellers, butchers and consumers may already be involved in; the level of existing public health regulation and enforcement; how enforcement takes place in practice; and the different ways that people respond to public health regulations.

## 2. Materials and methods

In this paper, we present findings from an ethnographic study in which major bushmeat markets in Sierra Leone’s second city–Bo–served as sites of participant observation over an extended period in 2022 and early 2023. We also carried out an extended in-depth focus group discussion to investigate the perspectives of three key public health stakeholders on bushmeat trading in the city.

Within Bo City, Sierra Leone, the majority of the trade in bushmeat takes place within two bushmeat markets. We initially experienced significant barriers when attempting to access bushmeat markets due to widespread mistrust of outsiders amongst traders which has resulted from what traders often describe as frequent harassment by parties responsible for enforcing laws and regulations in the areas of wildlife management and health and sanitation. Upon our initial visit to one market located on the periphery of the city, we were acutely aware that animal carcasses and bushmeat portions of some identifiable animals, including some species of monkey, were being moved out of sight. When we attempted to explain the purpose of our visit and the scope of our research activities to a small group of traders, we were informed that they were not interested in participating in our study because they were worried we were there to “cause problems”. Our ability to observe trading activities and to speak to traders in this market remained difficult over the course of our research despite repeated visits, however in mid-2023 author TH was able to spend time in this market observing butchering, trading, and preparation of bushmeat.

While we have included some discussion of how these activities are carried out in the purpose-built market, the less formal market located in the centre of the city served as our main study site and is the main focus of discussion in this paper. We were more successful in connecting with traders who operate at this market by making use of connections with hunters that we had established previously, during participant observation of hunting activities in rural areas of Bo District. One of our Sierra Leonean authors (WL) first made contact with a trader via phone call and made arrangements to visit the market alone to discuss our research in the Mende language. We were aided in this initial entry by the goodwill created from our links to some of the traders’ home villages, where JJ and WL had participated in group hunting activities in the forest with local youths. Follow-up visits were arranged in which authors met with leaders of the bushmeat traders’ informal organisational committee, including the chairperson and deputy chairperson. In contrast to our experience at the other of the city’s markets, traders were more open to listening to our explanation of our research activities, which included a reading of our project participant information sheet and consent form in both the Krio and Mende languages, and consented to further visits and interviews with traders when appropriate.

To assess how the organisation of bushmeat markets impact the potential for zoonotic disease contract and transmission, this study focused on identifying: (a) the potential for zoonotic disease transmission that may arise from traders’ and consumers’ close contact with animal blood and fluids in the bushmeat markets; (b) how market organisation and trading practices create opportunities for human contact with animal blood and fluids; (c) where responsibility lies for hygiene regulations in the sale of bushmeat and the enforcement of public health measures; and (d) traders’ knowledge of and level of compliance with public health regulations and any other hygiene measures taken by traders. We utilised an ethnographic approach to research that was based on extended participant observation alongside interviews that ranged from informal discussions to formal recorded interviews. JJ and WL observed traders, hunters, and marketgoers as they went about their regular business over more than 8 months. The research was inductive–we went to the markets to learn about the trade and consider risks for zoonotic disease transmission and followed traders’ practices to see what was happening. During these observation activities, we took preliminary handwritten fieldnotes that described in detail the setting and events that unfolded whilst we were present, which were written up soon after the event. We also asked participants questions relating to their actions. Once trust was established, we selected some traders for participation in interviews designed to gain a deeper knowledge of their business, including how meat is sourced and brought to the market, whether public health measures are in place and enforced, and the importance of their activities for their livelihoods. We conducted two in-depth interviews with traders that lasted for approximately one hour. These interviews were useful in confirming some of the issues raised in the numerous informal discussions we had had with traders previously. Issues raised in these interviews were also discussed with other traders during later participant observation sessions. By visiting on repeated occasions and talking to many different people we triangulated the findings and ensured we had reached saturation. In addition, we conducted a focus group with three officials working within the Department of Health and Sanitation at the Ministry of Health in which questions focused on responsibility for the formulation and enforcement of public health measures. We did not apply a theoretical framework to the findings but instead, working in the ethnographic tradition, we have used the article to generate theoretical and empirical insights through the findings and in conversation with the literature.

Ethical approval was obtained from the Sierra Leone Ethics and Scientific Review Committee (approval number 002/08/2022) and the Durham University Anthropology Research Ethics Committee (reference number ANTH-2022-04-22T14_10_41-qfvj43). Potential participants were given the opportunity to consider whether or not they wanted to participate before giving written and/or verbal consent. We obtained signed consent from all participants whom we approached in a formal work environment (e.g., Health and Sanitation officials) and verbal consent for all other participants. Verbal consent was documented in writing by a witness or by the person who obtained the consent. Consent for observations and informal conversations were obtained informally: individuals were informed verbally about the study’s details, benefits and potential risks, and any questions were discussed. Verbal consent to participate was documented in writing in the researchers’ field notes. Recruitment of participants took place between the 6^th^ of September 2022 and the 12^th^ of June 2023. Recorded interviews and focus group discussions were transcribed and translated into English. Analysis of transcripts and ethnographic field notes was carried out using NVIVO software.

## 3. Results

During most of our visits to the market in the centre of Bo which acted as our main study site, the gender makeup of the traders present was exclusively female. On a few occasions, a male friend or family member of one of the traders could be seen sitting on one of the benches underneath the market shelter. Many of the traders were young women, and some of them were single mothers whose participation in the bushmeat trade was their primary means of feeding their families: *We are mostly single mothers*, *and we are facing a lot of constraints here*. *It is difficult to get money to take care of our children*. The exclusively female gender makeup of the bushmeat traders stands in contrast to the gender makeup of the hunters encountered during our research, who were exclusively male. Interestingly, in the purpose-built bushmeat market on the periphery of the city, on some occasions we witnessed males participating in the butchering of larger animals such as bush cows and bush pigs. Within the market’s ‘kitchen’–an area outside of the main building that was not built as part of the main construction–it was exclusively women who could be seen roasting and steaming meat as a means of preservation. Both males and females could be encountered purchasing bushmeat at the markets.

Customers came to the markets to buy bushmeat for their household consumption, to resell in individual stalls elsewhere, or to prepare for public consumption at local restaurants. Some of the species commonly sold include deer, duiker, porcupine, bush pig, cane rat, and monkey, however the availability of different species varies each day and by season depending upon what can be sourced from the hunters who supply the markets. Monkeys were commonly cited to be the most desirable animal for consumption while cane rats and duikers were stated to be preferred when monkey meat is not available or is too expensive. Technology is increasingly used by traders to both source meat from hunters and advertise meat to customers. As illustrated by an ethnographic account included at a later point in this paper, traders sometimes receive photos from hunters of the bush animal killed before negotiating a sale and transporting the meat. Traders also sometimes take photos of the different meats available at markets and send them to their customers as a way of advertising.

### 3.1 Opportunities for human contact with animal blood and fluids

The bushmeat markets we studied were environments where hunters, traders and customers converged to transact over wildlife. Traders reported that the majority of bushmeat sold in Bo’s markets originated in rural locations in Bo District and neighbouring Pujehun District. Hunters often travelled to the markets with their catch to sell it to the traders who would then butcher the animal and resell its meat to the public. Most bushmeat sold in Bo supplies urban demand within the city. Sometimes bushmeat is bought by customers visiting from other locations such as Freetown, however visitors from the capital seeking to buy bushmeat often do so at the roadside during the return journey. The local bushmeat trade does not appear to be integrated with international markets. International transport of bushmeat sold in Bo was not raised by any participant at any stage.

During transport to markets, animals were commonly bagged in rice sacks or backpacks. Hunters, motorcycle taxi operators, and traders came into contact with fluids while packing or unpacking the animal, or touching a contaminated bag afterwards. Bagging animals not only helped to contain fluids, but also to hide the contents from law enforcement officers who may be encountered at checkpoints. Encounters with officers attracted complaints from both hunters and traders, who frequently bemoaned having to pay bribes even when the species being transported were not subject to any regulations restricting their hunting or sale. There is therefore a chance that officers or enforcement agents might also come into contact with the blood and fluids of animals being transported during an inspection or in the event of confiscation.

**Fig 2:**
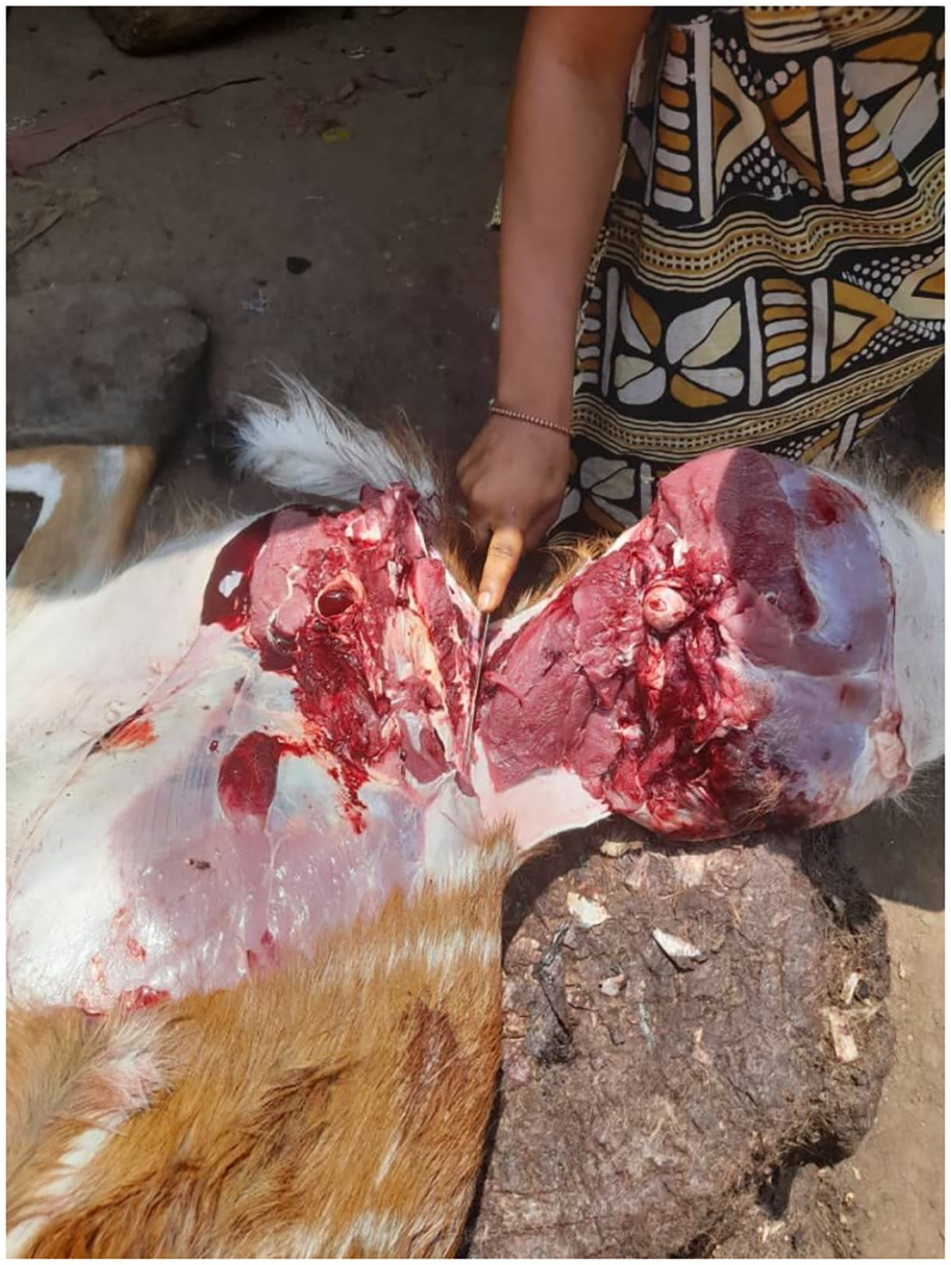
Photo showing the butchering of bushmeat by a trader in a bushmeat market in Bo City.

At the markets, there was frequent contact between humans and non-human species, including through the handling, butchery (see Fig 2), selection and packaging of carcasses and meat by hunters, traders, and customers. When bushmeat arrived at the markets, traders vying to buy the meat from hunters would often handle the meat by hand to inspect its condition. This was done without gloves or protective equipment, even when the carcass was oozing blood.

During the sales process, both traders and consumers directly or indirectly came into contact with animal blood and fluids. Traders encountered the blood, tissue and bodily fluids from wildlife many times a day on an everyday basis. The potential for disease transmission from wildlife to humans (either directly or indirectly) was therefore observed to be high. Possible dimensions for direct transmission included direct handling of bushmeat (including blood and body fluids) and the butchering of animals in the open by all vendors. Indirect transmission routes including inadvertent splattering of blood during butchering; walking through blood and other animal remains; splattering of contaminated water used for washing meat, hands and equipment during disposal; and the circulation of contaminated material objects including knives, trays, and money.

**Fig 3:**
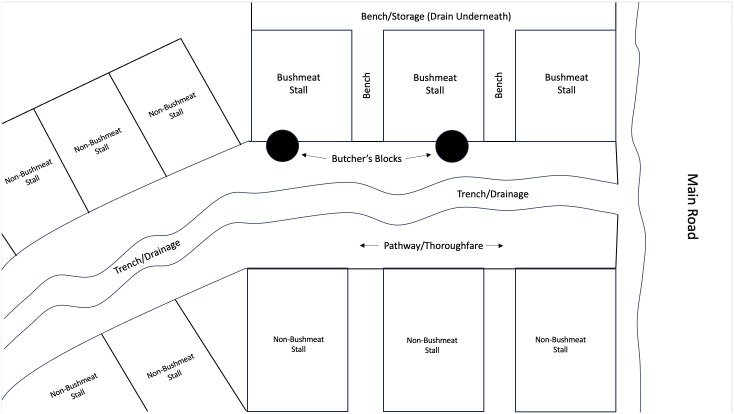
Diagram showing the layout of Toabu market, Bo.

**Fig 4:**
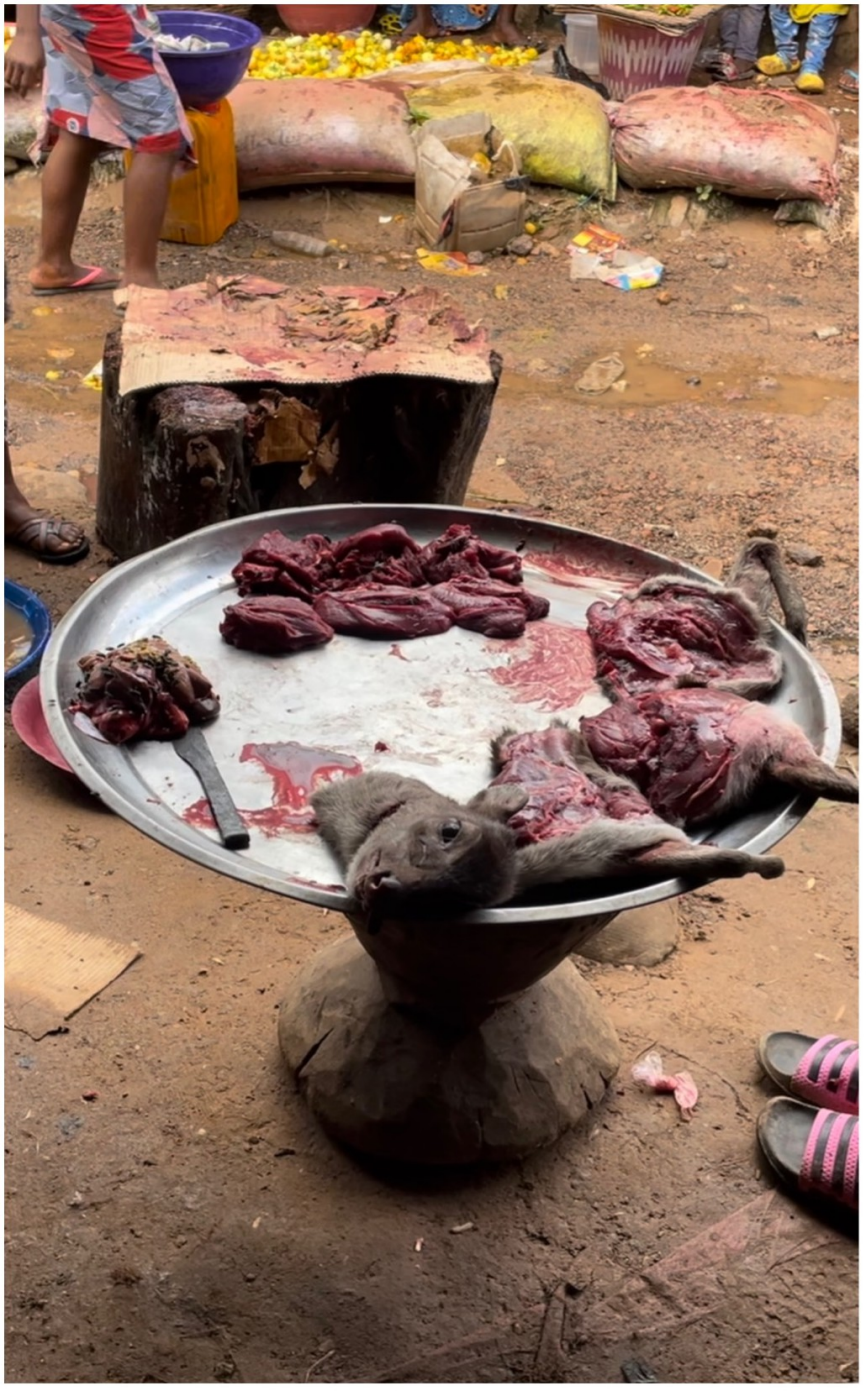
Butcher’s block and display platter with fly-covered meat next to the busy public walkway.

While the market that is located on the periphery of Bo city is located in a purpose-built structure complete with a dedicated butchery area, the market that served as our main study site took place under informal shelters arranged either side of a busy unpaved walkway in the city centre. While public water sources and toilets were available in the newly built market, similar facilities were not available in the city centre market, requiring traders to bring water from sources outside. Our focus on this informal market space is important because most bushmeat in Sierra Leone is sold in these kinds of informal settings rather than in dedicated markets where bushmeat is exclusively sold. The purpose-built location in Bo is a rare exception. As shown in Fig 3, the stall areas underneath the shelters are separated by wooden benches which also run along the back of the structure. At the front of these stalls, meat that has been butchered and arranged into individual portions–for example, individual legs, arms, or ‘lumps’ of meat–are displayed on metal platters in view of the potential customers walking by. The meat displayed on the trays is typically uncovered and is therefore frequently covered by large green flies (as shown in in Fig 4). We found that people commonly associate these types of flies with dirty areas, such as areas around latrines. The lack of protection from flies, which are potential hosts of pathogens, increases the possibility of the contamination of meat.

**Fig 5:**
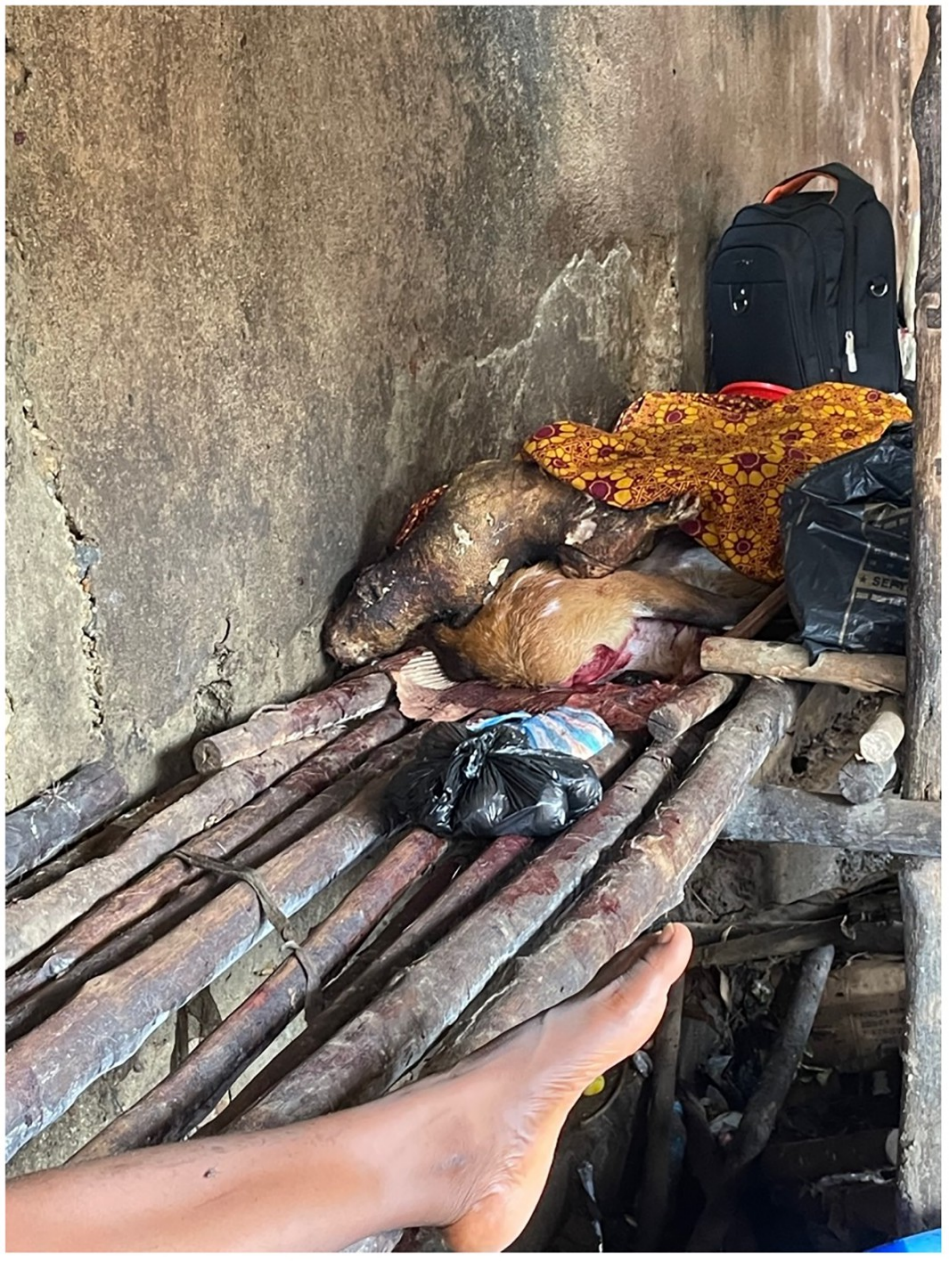
Roasted cane rat and unbutchered duiker stored on a bench at the rear of the market shelter.

Carcasses that have not yet been butchered or have been partly butchered were stored on benches at the rear of the shelters, sometimes covered in cloths and sometimes left open to the air and flies. Carcasses of different animals and different species are stored on top of each other, even when leaking blood and fluids (see Fig 5). On some days, greater numbers of animals and greater varieties of species are stored on the benches. Despite benches being stained with blood and fluids, people sit on these benches when they have dried. Located directly underneath this rear bench is a gutter in which many maggots can be found, particularly during the dry season when the daily temperatures are higher. When the traders are ready to butcher the carcasses, they are brought to the front of the structure–on the edge of the walkway–where the traders butcher meat on large cylindrical butcher’s blocks made from tree trunks. Blocks are usually topped with a sheet of cardboard that is used all day, for all animals. The traders use axes to chop through bones and knives to slice meat and to separate the skin and cartilage from the flesh.

Some of our key observations were to identify potential routes of indirect contact with animal blood and fluids by market goers and market traders not involved in the bushmeat trade. During the butchery process, when axes are raised and brought down, animal blood and fluids are sprayed onto passers-by, neighbouring market stalls, and the traders themselves. We regularly witnessed animal blood and fluids being sprayed on vegetables such as garden eggs (African aubergines/eggplants), chilli peppers, tomatoes, beans, and fruits, potentially causing cross-contamination of fresh produce. While we observed pedestrians being sprayed with fluids and apparently noticing that they had been sprayed (for example by looking down at their clothes or bodies and then to the butcher/trader), we did not witness anybody complaining or reacting angrily. Nevertheless, the fact that fresh produce and members of the public are regularly sprayed with the fluids of wild animals when walking through the centre of a major city should be seen as a significant public health risk. Furthermore, during rainy season, we observed that the centre of the walkway along which market goers walked had formed a trench into which the runoff from the butchery process ran (visible in Fig 4), creating a further reservoir of potentially contaminated liquid which often splashed onto the clothes and bodies of passers-by. On one occasion, we witnessed a bread vendor drop a bank note into the drainage trench, pick it up, and continue selling bread by hand without washing his hands.

Once they have been butchered, some parts of the animal including the intestines are cleaned by hand in bowls of water. As noted, water must be brought from outside, and is therefore kept in storage containers. Many containers are open-topped and therefore susceptible to contamination during the butchery process. We regularly witnessed traders washing meat in the bowl before passing it by hand to other traders who would take it (again by hand) and place it on metal display platters. The washing of hands and butchering equipment (e.g., knives, axes, and rags) was noted to be infrequent and we did not observe vendors using soap. The same water used to clean butchering equipment for one animal was observed to be used for all almost all butchering. Water used for washing would be discarded either in the trench in the middle of the walkway or in the gutter at the back of the shelter, underneath the bench where carcasses were stored. Traders would often handle meat with their hands before handling money or going back to using their smart phones. On some occasions, we witnessed paper banknotes being placed on the display platters alongside meat and blood, representing a further means of potential indirect contact with animal fluids. In addition, consumers were observed to come into direct contact with bushmeat by handling the meat when selecting the portion that they wished to buy. As many of the traders are mothers, it was unsurprising that we often encountered young children within the market setting. We observed children joining their mothers and care givers at the market after school. These children not only engaged with customers, but also handled meat, fetched water, and swept the market floor. Younger children were often allowed to play on the floor of the shelter on their hands and knees, in an area repeatedly splattered by animal blood and fluids, as well as by water used to wash meat. We witnessed children and babies playing on the floor underneath trading tables around drops of animal blood and fluids not only in the less formal city centre market, but also in the purpose-built market located on the outskirts of the city. Sometimes children needed sanitary attention from their mothers but due to their need to attend to customers, these mothers could not attend to their babies as urgently as required. We witnessed how a mother could not attend to her child who had soiled herself and was crying for over fifteen minutes with flies on her body. Despite other traders calling her to take care of the baby, all she said (in Krio) was “*wait while I make money to take care of you*”, revealing the complex balance of responsibilities traders must face in taking care of children while providing for them financially. Even after cleaning the child, the woman did not wash her hands but rather continued with her business.

**Fig 6:**
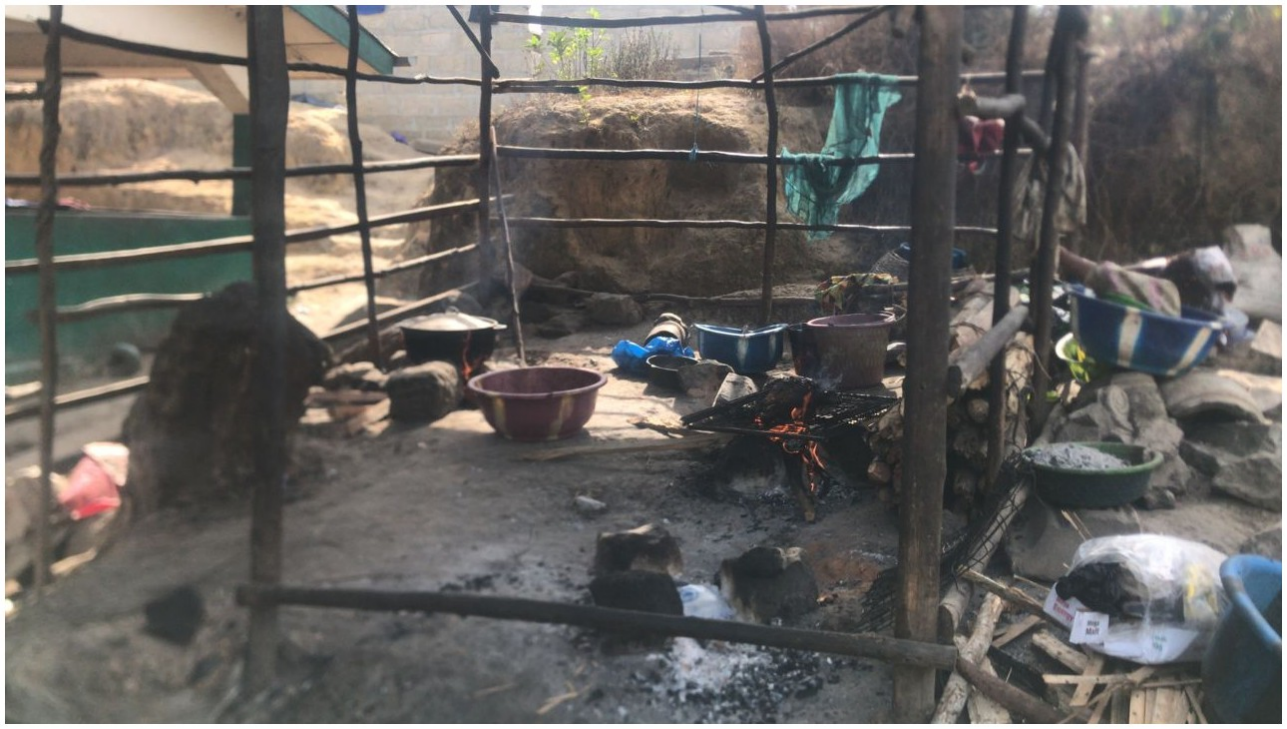
Makeshift ’kitchen’ area at the rear of a purpose-built bushmeat market in Bo City.

As noted above, the purpose-built market has a ‘kitchen’ at the rear which was not part of the original construction. Rather, it is a makeshift structure made with sticks and sheet metal roofing (see Fig 6). It has four separate stoves each made from three stones arranged in a triangular shape. Smoking, steaming, and drying meat is predominantly done by two women who have developed expertise over time. The two women are present at the market throughout the day to maximise their custom. They report regularly experiencing health issues including headaches, eye problems, and chest pains which they believe to be caused by the high levels of smoke produced during their activities. The two women are paid for their services by the other traders and sometimes by customers who require their services in preparing meat bought from the market.

Neither of the women use any type of protective equipment despite clear sanitation challenges. They note that the market construction project did not make adequate provision for waste disposal despite numerous traders and community residents raising concerns. Waste produced by traders and butchers in the market, as well as by the women who process the meat at the rear kitchen, is dumped in a nearby swamp by local boys for a sum ranging from two to five Leones. Dumping of this waste at the swamp creates environmental hazards which could expose community members to zoonotic disease risks. Therefore, while this market has water access and a dedicated butchery area away from the public, inadequate waste disposal and non-use of protective equipment are common issues across the two study sites.

Our findings show that the organisation of these bushmeat markets, coupled with the behaviour of traders in the handling, butchering, and marketing of bushmeat creates a high-risk environment for the transmission of both zoonotic and non-zoonotic diseases. Traders have prolonged, repeated contact with animal fluids without protective measures. Butchering practices and waste disposal bring the public and other vendors into contact with these fluids. Children are also present and are potentially at risk of exposure to zoonotic disease. As will be clear in the next section, children were not specifically mentioned by stakeholders as playing a role in the bushmeat trade or as a group at risk of zoonotic disease transmission. Animals were brought to the market dead. In the next section we will also discuss how this means that they were not subject to the types of disease monitoring or antemortem inspections that take place by veterinarians and sanitation workers at slaughterhouses, which is a further significant cause for concern.

### 3.2 Responsibility for regulation of the sale of bushmeat and the enforcement of public health measures

In Sierra Leone, the enforcement of public health regulations in the slaughter and sale of meat (including bushmeat) falls under the remit of various different public institutions. The Ministry of Agriculture is responsible for live animals and conducts antemortem inspections on animals scheduled to be slaughtered at slaughterhouses, but once these animals are killed, they are deemed to have become food and thereby become the responsibility of the Ministry of Health and Sanitation. The Public Health Ordinance 1960 is still in effect despite being enacted prior to independence [30]. While the ordinance deems any (domesticated) meat or carcass slaughtered outside a public slaughterhouse to be unfit for human consumption, it includes provisions that allow the slaughter of sheep and goats by Muslims during the festivals of Bairam and Ramadan, and the killing of “any wild animal or game shot, trapped or otherwise killed in the bush for food for human consumption” (Public Health Ordinance, 1960, Schedule 115 (1)) [30]. Unlike for domesticated animals, the killing of wild animals outside of the market does not therefore automatically deem their meat to be unfit for human consumption. The Ministry of Agriculture does not have oversight of the slaughter of bushmeat, as animals are typically killed long before they arrive in the city, when they are caught in traps, caught in nets, caught by dogs, or shot. A public health officer expressed his concern over the potential risks created by this lack of oversight:

*“[For] some people [hunting and trading bushmeat] is their livelihood but to me [with] any meat that is not inspected*, *as we all know*, *that we have zoonotic diseases*. *Most people get sick as a result of consuming meat from animals taken from the bush*. *So*, *we are afraid [of] zoonotic diseases particularly when these meats are not inspected before consumption”*

—Ministry of Health and Sanitation Officer.

As a representative of the Ministry of Health, we acknowledge the stakeholder’s role may shape how he perceives and discusses the issue with researchers. Nevertheless, despite a mandate to ensure that all meat, whether it comes from wild or domesticated animals, is sound, safe and in a good condition for people to consume, it was clear that the main focus of the local department of public health and sanitation was on the slaughterhouse. Despite their apparent focus on the safety of domesticated meat, officers explained to us that they still perform a role in ensuring the safety of bushmeat when possible. Health and sanitation officers sometimes visit bushmeat markets alongside council workers to advise on whether meat is fit for public consumption. However, it was not made clear whether these officers have received the training required to adequately fulfil their mandate.

Local authorities including local chiefs, section chiefs and paramount chiefs are engaged to summon individuals who are believed to have violated the Public Health Ordinance, aiming to address these issues at the local level initially. Animals deemed to be unfit for sale to the public may be seized and destroyed. Seized animals are sometimes disposed of in public view, in the presence of the media, the community, the council, the police and the owner of the meat, who is given a certificate of destruction. The Health and Sanitation officers we spoke to noted that sometimes members of the local community may come to the dumping ground to dig up the animal for consumption, so to discourage this they spray chemicals over and inside animals before they are buried. When asked whether they believed market sellers of bushmeat try to avoid contact with animal blood and fluids, they said that traders lack the knowledge or expertise to protect themselves and noted that they–as government officers–may have a role in providing education. However, it was clear that their activities that related to bushmeat were fairly limited, especially compared to involvement in inspection and oversight of domesticated meat from the point of slaughter.

In summary, the enforcement of public health regulations relating to bushmeat is currently done through the collaboration of a range of institutions including the Ministry of Health and Sanitation, the City Council, the police, and local authorities. Bushmeat receives much less attention from the Ministry of Health and Sanitation than domesticated meat. It is important to note that due to much improved rural-urban transport linkages and greater market access, the food systems that supply both domesticated and wild meat to urban markets have fundamentally changed in the time since the systems for the management of the slaughter and sale of meat that were set up during the colonial period. It may therefore be necessary to re-evaluate the appropriateness of these systems for the 21^st^ century.

Within the current system, health and sanitation officers occasionally provide health-related advice to other partners that collaborate on enforcement, and sometimes join partners including the city council in inspections of bushmeat markets. However, the frequency of inspection activities is not clear, especially when compared to the daily inspections carried out in the slaughterhouse and at the city’s main market, where bushmeat is not sold. The collaborative nature of enforcement also serves to make it unclear which enforcement agents visit the market most regularly. In the next section, we explore the experiences and perceptions of bushmeat traders, shedding light on how the behaviours of some enforcement officers may have implications for public health.

### 3.3 Knowledge of and compliance with public health regulations by bushmeat market traders

Throughout this study, traders and consumers took few if any steps to reduce potential risks of disease contraction and transmission in the bushmeat markets, which could threaten public health safety. Traders’ responsibilities under the law to abide with public health measures sometimes came into conflict with their responsibilities to their families to maximise income, as discussed below. Traders described four public health measures, which were: (1) not selling meat that is starting to rot; (2) using gloves when butchering and handling bushmeat so as to prevent contact with the blood and other body fluids; (3) using mosquito nets to cover butchered meats to prevent flies (which are potential hosts of pathogens) from sitting on the meats; and (4) the use of face masks during the COVID-19 pandemic. Nevertheless, the effectiveness of public health measures appeared to be extremely low. A breach in regulatory frameworks could lead to the confiscation of meat by the health and sanitation officers, however vendors were observed to rarely follow these public health guidelines. This was apparently due to the regular practice of bribing enforcement officers by traders who were deemed to have committed an infraction of regulations. One market trader explained as follows:

*‘‘[We] were told not to sell bushmeat that is starting to rot*, *therefore*, *when the sanitation or police officers come and inspect and see us selling meat that is starting to rot*, *they will seize it*, *but I normally have to pay them off to allow me [to continue to] sell my meat’*

—Bushmeat Trader, Bo City.

Another trader described the same ability to get around regulations by paying bribes:

*“Sanitation [officers] come to look at how clean the place is*, *if we are wearing gloves*, *if we are covering the meat from the flies … [When] the sanitation people come they see that there is no covering*, *and when the meat has been caught in the trap and left there until it has become rotten*. *They take it away and say they are going to bury it*. *One time they came and took a whole animal away so they could bury it and cover it with chlorine*. *Sometimes when they see the meat is not covered*, *instead of them taking it away we can negotiate and give them some cash*. *When we do this*, *they allow us to keep it and keep selling it”*

—Bushmeat Trader, Bo City.

Perhaps because of lax enforcement of health and sanitation measures, when traders are left with unsold meat at the end of the day, they are likely to try to protect their investment by finding a way of still making money from it. The following conversation with a trader who had agreed to purchase an animal via instant messaging that morning shows the concerns that traders experience when they are left with unsold meat later in the day:

*“The reason this one here [a bush pig] has lost its form*, *is that they brought it late*, *so the price is also going to fall*. *I’m not going to make profit out of this because they should have brought it this morning*. *They also brought it on top of the car under the sun*, *which made it lose its form*. *It is strong [i*.*e*. *tough] now–difficult to slice–so people aren’t going to buy at the expected price*. *I bought this whole bush pig for 1*,*300*,*000 Leones (approx*. *$81*.*25 USD) and it has spoiled already*. *One leg would usually sell for 250*,*000 Leones (approx*. *$15*.*63)*, *but I won’t get that price now that it has spoiled”*

—Bushmeat Trader, Bo City.

At the time of our conversation, she and other traders had begun to butcher the animal and a strong smell of decay had filled the market shelter. When we returned to the market a few weeks later, the trader told us that she had given the meat on credit to a friend who owns a restaurant, who would pay her back later from the proceeds of the bushmeat soup she would sell to the public. She explained that she had considered steaming the meat and bringing it back to the market for sale the next day, but she would have struggled to make any profit that way, so had given it to her friend instead which would secure her a profit of 200,000 Leones (approx. $12.50 USD). While this makes sense from an economic perspective, from a public health perspective it is a concern. Supplying local restaurants with meat left over at the end of a long, hot and humid day of trading that is on the verge of rotting or has even begun to rot presumably poses significant risks to public health. Trefon [12] notes that Congolese bushmeat traders sometimes inject formaldehyde into the carcasses of unsmoked animals to preserve them, however did not observe any instances of traders using formaldehyde to preserve meat during our study in Sierra Leone.

As the above quotes show, it was the opinion of the traders themselves that they have knowledge of public health guidelines but that these are generally not followed. Meat that was starting to rot continued to be sold to the public despite potential punishment because public health and sanitation officers could be paid off, allowing sellers to continue to trade. In addition to the lack of a real incentive to abide by regulations due to lax enforcement, some hygiene measures also come with a financial barrier to compliance. For example:

*‘‘[Some] of these items they said we should use to prevent us from direct contact with the meat*, *for possible disease contact and transmission*, *are not provided to us … Most times when they come and see that our [meat is] not covered with nets*, *they will seize them*, *[and] not until we pay them off [do they] allow us to sell*. *And the profits we get from the trade is too small that we cannot afford to buy the items for ourselves*, *but if anyone could provide these items*, *we can use them”*

—Bushmeat Trader, Bo City.

One trader focused on gloves when describing her difficulties in complying with public health regulations:

*“They came and said during the pandemic that we should use gloves to protect ourselves from diseases*, *but they don’t give gloves [to] us*, *so we have to buy them for ourselves*. *If they were to provide [them] we would make use of them but now we don’t do it”*

—Bushmeat Trader, Bo City.

Traders are aware of various public health regulations but widespread non-compliance persists due to ineffective enforcement, a lack of means to comply with regulations, and the ability of lawbreakers to bribe enforcement officers, creating an environment where regulations are often disregarded. This raises the question of how market conditions may be improved to address the pronounced health risks that current practices in bushmeat markets pose to market sellers, market goers, and the wider public, as discussed in earlier sections. With effective enforcement so obviously lacking, the introduction of new laws and regulations does not appear to be a credible solution. Bushmeat sellers express a willingness to make use of protective equipment such as gloves and net coverings, but they note they are unlikely to be willing or able to purchase this equipment themselves due to financial barriers. The provision of suitable equipment including gloves, net coverings, and soap or sanitiser may be an effective strategy that has already been employed temporarily during the COVID-19 pandemic.

## 4. Discussion

Widespread disregard for basic sanitation and hygiene–such as not covering meat, cleaning surrounding areas or washing hands and equipment–suggests that enacting behaviour change to reduce the risk of the emergence or re-emergence of zoonotic diseases would be particularly challenging. Based on his research in bushmeat markets in the Congo Basin, Trefon [12] says:

From a public health perspective it is difficult to campaign for behaviour change with regard to invisible infectious diseases when very poor people cannot respect even basic sanitation recommendations. Inadequate public service provision, lack of funding, chaotic management and practically non-existent awareness about basic hygiene are some of the factors that translate into an overwhelming disregard for biosafety.

These factors appear to be common across both the Congolese and Sierra Leonean contexts, as do the inadequate or exploitative actions of law enforcement, public health, and environment representatives. As described above, it is common for public health and sanitation workers to look to extract fines or bribes from bushmeat sellers who have little choice but to pay. In many cases, the enforcement of laws and regulations is secondary to individual gain, as representatives who have received a bribe from sellers may allow them to keep unsafe meat and to continue selling it to the public. In the Central African context, Trefon [12] notes that public health or environment representatives would most likely be more interested in collecting an informal tax or bribe from sellers at urban markets rather than in making sure that the official health and sanitation norms–which they don’t have the means to control–are respected. As Saylors et al. [6] note, perceived inconsistent implementation and lack of fairness in application of bushmeat regulations is a source of conflict between regulatory authorities and vendors, making it challenging for public health messaging to be taken seriously by actors who do not trust those responsible. In Sierra Leone, the existing public health ordinance has been in place since the colonial period. Updating this ordinance to take account of the realities of the modern-day bushmeat trade would likely improve the practicality and legitimacy of the regulatory framework in the eyes of those working in the trade. Furthermore, if the implementation of an updated and realistic legal framework were to be accompanied with dedicated public awareness or community sensitisation campaigns that could equip market sellers, hunters, and consumers with improved knowledge of their rights and obligations, they would be rendered less vulnerable to the apparently widespread exploitative behaviour of enforcement agents. However, as highlighted by Saylors et al. [6], it is important to develop an appropriate engagement strategy to ensure effective community buy-in.

The conditions described here suggest a significant risk to public health posed by the organisation of bushmeat markets and some of the practices used by traders, for example in the butchering of meat and in waste disposal. However, it is not likely that prohibition would do anything other than push the market away from its current location to one that is even less formal and less organised, making regulation and oversight more difficult. Indiscriminate market bans would disrupt the informal food systems on which millions of people depend [28]. Elsewhere, Petrikova et al. [31] argued that banning wet markets would likely disadvantage human health because it would remove an important source of protein for many populations. Bonwitt et al. [32] similarly found that the bushmeat ban enacted in Sierra Leone in the context of the 2013–2016 West African Ebola virus disease (EVD) epidemic served to drive the bushmeat trade underground and out of sight rather than preventing the trade of bushmeat. This only served to weaken surveillance for future outbreaks, reduce food security for dependent communities, and diminish trust in authorities [32]. If wildlife markets are forced to operate clandestinely, regulations governing hygiene and animal welfare become harder to enforce, thereby increasing the risk of zoonotic disease outbreaks [28].

As LeBreton et al. [5] suggest, the use of bushmeat is likely to continue, so people should be encouraged to undertake hunting and butchering more safely for their own and their community’s health. Petrikova et al. [31] argue that improving hygiene in markets is an important step towards reducing zoonotic risk. While we agree that improving hygiene is vital, we are not in agreement with their suggestion that this might be achieved by regulating markets more stringently. Rather, the tension between traders and public health enforcement agents we identified in this study suggests that greater enforcement and regulation would not be the best way of improving market hygiene in Sierra Leone. Furthermore, some of the measures they suggest, such as outlawing sales of wild animals known to be of risk for disease spread, do not seem practicable or realistic in this context.

In our main study site, market structures have been developed over time without official planning or thought paid to how the layout impacts upon public health. Water must be brought from outside, discouraging regular hand washing. One trader explained that the market land and shelters are owned by two private individuals who collect rent from traders each month. They explained:

“*The money that we are paying is limiting the money we are making from our business*. *We are really constrained*. *If the government or any organisation could make a market like the one they have at Shellmingo*, *it would really help us*”

—Bushmeat Trader, Bo City.

However, as noted above, our main study site is not the only bushmeat market in the city of Bo. A second larger market exists on the periphery of the city, on the road leading towards the Southeast of the country and the Liberian border. In the past, this location was also the site of an informal market where bushmeat was traded by street vendors without adequate facilities for hygienic butchery or waste disposal. However, in recent years a market building was constructed which comprises a main market area and a dedicated space for butchery at the rear, in addition to water sources and toilet facilities. With the butchery area separate to the area where traders interact with the public, the opportunity for customers and passers-by to be splashed with the blood and fluids of wild animals is much reduced. The contrasting conditions between these two markets illustrates how the adequacy of appropriate facilities and sanitation infrastructure in markets contributes to zoonotic disease risk in these spaces.

Lucas et al. [19] state that PPE appears to be universally unaccepted by bushmeat vendors and posit that they would be unlikely to purchase PPE themselves or to use them should they be provided. Our findings confirm that vendors in Sierra Leone are unwilling or unable to purchase PPE themselves. However, they report having used PPE when they were provided to them during the COVID-19 pandemic, perhaps suggesting a greater willingness to use PPE than in other settings. During the 2014–2015 Ebola epidemic, handwashing was also more frequent, with veronica buckets containing chlorinated water positioned in many locations, including at the entrances to markets. Provision of PPE, hand washing facilities, and equipment such as fly nets could be components of a strategy aimed at encouraging voluntary compliance with safer practices. Such a strategy may produce improved conditions and reduces the public health risks associated with current practices. Engaging market traders’ and women’s associations is important for fostering behaviour change and improving public health conditions associated with current practices.

Our deliberate focus on two bushmeat markets in a single city was necessary given the ethnographic methods employed. However, we cannot therefore claim that our findings hold true across all markets in the country or in bushmeat markets elsewhere in the region. Nevertheless, as we have noted previously, our approach to studying these spaces is both novel and important because most bushmeat in Sierra Leone is sold in these kinds of informal settings. Future research aimed at assessing the generalizability of our findings in other contexts would provide valuable insights into the magnitude of the zoonotic risk posed by bushmeat markets more broadly.

## 5. Conclusion

Bushmeat is traded on a large scale in markets in the city of Bo. This ethnographic study sheds new light on how the characteristics and daily rhythms of the bushmeat market create opportunities for disease transmission to humans from wild animals. It shows that when we look beyond an ontological imperative that tells us that risk of zoonotic infection ‘must be’ the result of the knowledge, behaviours and practices of traders we gain a richer and more useful picture of the multi-dimensional nature of this potential zoonotic interface. Given that studies of bushmeat markets have often focused on traders and butchers and have rarely explored the risks they pose to customers, children, and members of the public, we believe this to be a key contribution.

Generally, the two markets that were the focus of this study remain sites at high risk of zoonotic disease contact and transmission. We do not seek to extrapolate the findings from this study of two markets in Bo to claim that these conditions are common in all markets across the whole of Sierra Leone. The distinct characteristics of different markets present unique opportunities for human-animal contact. However, our findings show how inadequate facilities and sanitation infrastructure in more informal bushmeat markets can create significant zoonotic disease risks. It is important to acknowledge these risks given that most of the bushmeat traded in Sierra Leone is not traded in dedicated bushmeat markets. We suggest that, rather than the ‘stick’ of stringent regulation and enforcement, a more realistic way of encouraging better hygiene practices amongst bushmeat traders may be to use the ‘carrot’ of providing better spaces for butchering and trading meat and providing traders with PPE and hand washing facilities. The answer may therefore be in improving conditions, for instance by improving market infrastructure and facilities that allow vendors to practice safer behaviours, reducing their personal risk as well as the identified risks posed to other market users. The construction of a new market building in the city centre–whether at the existing site or at a site nearby–may help to address the obviously significant public health risks that arise from open butchery of wild animals in close proximity to often crowded, heavy pedestrian traffic. This may also help to build good will between traders and authorities, moving public health strategies away from those focused on enforcement to one of voluntary compliance. Engaging with market traders’ associations and women’s associations could aid this transition.

This type of approach which recognises that the bushmeat trade is unlikely to go away contrasts with more coercive approaches to controlling the trade, such as the bushmeat ban enacted in Sierra Leone in response to the Ebola outbreak, and the stringent measures including outright bans that some have called for following the COVID-19 pandemic. Because previous attempts to enforce such measures appear to have failed, approaches that aim to encourage voluntary compliance with public health recommendations and the use of biosafe practices appear to be warranted.

## Supporting information

S1 File Semi-Structured interview guide for bushmeat traders. (DOCX)

S2 File Semi-Structured interview guide for government workers. (DOCX)

S3 File Relevant qualitative excerpts. (DOCX)
