# Supplementary material for: Human-animal entanglements in bushmeat trading in Sierra Leone: An ethnographic assessment of a potential zoonotic interface
Source: PLoS One. 2024 Mar 28;19(3):e0298929. doi: 10.1371/journal.pone.0298929 (PMC10977710; doi:10.1371/journal.pone.0298929)
Supplement: S1 File — (DOCX) [file pone.0298929.s001.docx]

**S1 - Guide for Interviews with Bushmeat Traders**

- What is it like being a trader these days?
- Do you do any other income generating activities in addition to trading?
- What types of bushmeat do you trade?
- Please could you tell me about how you source your bushmeat?
  - **Prompt:** From who and from where?
  - How would you describe your relationship with the people who you get the bushmeat from?
- Around how much bushmeat of each type do you trade each month?
- How does this number compare to the amount you traded in the past?
  - **[If applicable]** What are the reasons for this change?
- Do you have to follow any restrictions/regulations when trading bushmeat?
  - **[If yes]** When were these restrictions/regulations put in place?
  - Do people follow the regulations?
  - What is your opinion on these restrictions/regulations?
- Please could you describe the transport process from when you buy the bushmeat to when it arrives at market or the place of sale?
  - Is this process the same throughout the year?
  - How much does transport cost?
  - How long do you typically spend transporting produce?
  - How would you describe this process in terms of difficulty?
  - Has transport improved, worsened or stayed the same in recent years?
  - Prompt: Access to markets; time taken; cost of transport.
  - **[If applicable]** Have these changes in transport impacted your business?
  - What changes could be made to the transport situation to help your business?
- Do you only trade bushmeat or do you also trade other goods/produce?
  - **[If yes]** What other goods do you trade?
    - **Prompt:** Sale of other protein foods.
  - Where do you source these goods?
  - Where do you sell them?
  - Who buys these goods?
  - Have you traded all these goods for the same amount of time or are some more recent?
  - How does trading these goods compare to trading bushmeat?
- Do you think you will continue trading about the same amount of bushmeat in the future (5 years/10 years)?
  - Why do you think the amount will change?
- Before we finish this discussion, would you like to make any further comments?
