# Supplementary material for: Human-animal entanglements in bushmeat trading in Sierra Leone: An ethnographic assessment of a potential zoonotic interface
Source: PLoS One. 2024 Mar 28;19(3):e0298929. doi: 10.1371/journal.pone.0298929 (PMC10977710; doi:10.1371/journal.pone.0298929)
Supplement: S2 File — (DOCX) [file pone.0298929.s002.docx]

**S2 - Topic Guide for Interviews with Government Staff**

- To begin, please could you tell me about the ways in which people in Sierra Leone are involved in hunting animals?
- In your view, what is the importance of these activities in rural areas?
  - Prompt: livelihoods, nutrition, culture.
- Do you think that in the future there will be the same level of participation in hunting activities, or do you think this will change?
- In your experience, do you think that the ways in which people hunt animals have changed over the years?
- Please could you describe the process in which bushmeat is traded?
- Do you think that this process has changed over the years?
  - In your view, what were the reasons for these changes?
- What is the importance of bushmeat for nutrition?
- Do you expect that bushmeat will become more or less important in the future?
  - In which ways do you feel that it will become more or less important?
- Have any alternative sources of protein become available in recent years?
  - What do you think were the reasons for these alternatives becoming available?
- Is hunting an activity that should be encouraged?
  - For what reasons do you think hunting should be encouraged/discouraged?
- Are the authorities trying to influence hunting behaviours in the country?
  - How are the authorities trying to influence hunting behaviours?
  - Do you expect that these initiatives will be successful?
  - In your opinion, how could the effectiveness of these types of initiatives be improved?
- Before we finish this discussion, would you like to make any further comments?
