# Supplementary material for: Human-animal entanglements in bushmeat trading in Sierra Leone: An ethnographic assessment of a potential zoonotic interface
Source: PLoS One. 2024 Mar 28;19(3):e0298929. doi: 10.1371/journal.pone.0298929 (PMC10977710; doi:10.1371/journal.pone.0298929)
Supplement: S3 File — (DOCX) [file pone.0298929.s003.docx]

**S3 – Relevant Qualitative Excerpts**

**Date:** 4^th^ November 2022

**Place:** Health and Sanitation Department, Bo City, Sierra Leone

**Time:** 9am

**Number of participant (disaggregated by sex):** M2F1

**Ethnicity:** Unknown

**Category/Cadre:** Government Key Stakeholders (Health and Sanitation Department)

**Interviewer:** JJ and WL

| Ministry of Health and Sanitation Officer | *“So, that’s why [my colleague] said we only have control over the [animals] in the slaughterhouse but the [animals] they are taking from the wild, they have a special market for them, and that market is being supervised by the council and the local authorities within the community”.* |
| --- | --- |
| Ministry of Health and Sanitation Officer | *“It’s only us that can tell council that this meat is not of good status to the community, [and] then Council needs to take the necessary actions. But if we advise [and] then those who should implement the law fail to implement [it], then it becomes a challenge”.* |
| Ministry of Health and Sanitation Officer | *“[Sometimes] we can go there to give advice because the manner in which those animals are exposed to the public, it is not good, so we advise them [but] when people are getting their livelihoods [in this way], it is very difficult. But we are trying our own part because we are thinking about the hazards behind it”.* |
| Ministry of Health and Sanitation Officer | *“[Our] own work or our mandate is to [focus on] food animals, I mean livestock, not wild animals … [It’s] not in our ordinance to inspect wildlife because they are already killed before they bring them, so we don’t know … But the only thing since people are eating them, they are food, and it’s our mandate to inspect foodstuffs especially those sold to the public, so we inspect if they are maybe about to go bad, to be decomposed or so, we will seize and destroy [them]. Also, we go around to advise them, we go around to see the soundness, but we don’t focus on them because it’s not food animals, [the] livestock, it’s wild animals”.* |
| Ministry of Health and Sanitation Officer | *“But if it is for, like we are talking about now the animals, if we realise that they are not safe, we dispose them in the public view. We invite the media, the community, the council, the police and the owner of the meat. We invite them all, we take them to the dumping site in the public view then we give them a certificate of destruction”.* |
| Ministry of Health and Sanitation Officer | *“But sometimes even when we are going for destruction, we may have other community people coming to dig out the animal. So, what we do, we have chemicals that we use, we dig and spray all the chemical inside, we deposit the animal then we cover [it]”.* |
| Ministry of Health and Sanitation Officer | *“It’s because of the knowledge, [bushmeat traders] don’t have the knowledge, the expertise. They are not protected”.* |
